# Postoperative gastric dilatation causing abdominal compartment syndrome

**DOI:** 10.1186/1749-7922-3-7

**Published:** 2008-01-31

**Authors:** Ahmad Mahajna, Sharon Mitkal, Michael M Krausz

**Affiliations:** 1 Department of Surgery A, Rambam Medical Center, and the Bruce Rappaport, Faculty of Medicine, Technion-Israel Institute of Technology, Haifa, Israel

## Abstract

**Objective:**

To study the effect of postoperative gastric dilatation on intra-abdominal pressure (IAP).

**Design and setting:**

Single case report from a primary teaching hospital.

**Patients and methods:**

A 72-year-old woman demonstrated a sudden respiratory and cardiovascular collapse following resection of a retroperitoneal sarcoma. This collapse was caused by abdominal compartment syndrome due to gastric dilatation.

**Results:**

The patient was re-explored, an enormously distended stomach was found with the nasogastric tube situated in a small sliding hernia which prevented drainage of the distended stomach. Re-positioning of the nasogastric tube, allowed the decompression of the stomach and the patient's condition immediately improved.

**Conclusion:**

Acute abdominal distention following major abdominal surgery may result from acute gastric dilatation, leading to oliguria and increased airway pressures. Untreated gastric dilatation can cause abdominal compartment syndrome.

## Introduction

Abdominal compartment syndrome (ACS) is defined as an increased intra-abdominal pressure (IAP > 20 mmHg) in combination with single or multiple organ dysfunction which was not previously present [1-3]. This condition affects multiple organ systems in graded fashion [4]. Early identification and abdominal decompression are essential in the management and treatment of this difficult situation, otherwise, it leads to multiple organ failure and ultimately, death [5]. Increased intra-peritoneal volume conditions are the most common source of elevated IAP [6,7]. Extrinsic compression of the abdomen by burn eschars [8], pneumatic anti-shock garments [9], tight abdominal closure [10], massive volume resuscitation for any reason, and inflation of the peritoneum by CO2 in laparoscopic surgery can also lead to increased IAP [11]. We present a rare case of abdominal compartment syndrome caused by postoperative gastric dilatation in 72-year-old woman.

## Case presentation

A 72-year-old woman was addmitted for elective resection of retroperitoneal sarcoma. The diagnosis was done by CT scan and pathological confirmation by explorative laparotomy 3 weeks before in another institution. At operation complete resection of the tumor was performed. Following surgery the patient was admitted to the intensive care unit. On arrival she was stable hemodynamically, her blood pressure was 135/80 mmHg and heart rate was 95 beats/min. During the first day the patient was mechanically ventilated on Fio2- 0.4, Vt- 600 ml, and a rate of 12/min. The peak airway pressure was 21 cmH2o and the mean airway pressure was 10 cmH2o. Her blood gases were within the normal range.

After 24 hours, the patient was extubated. Following extubation, her abdomen became gradually tenser and distended, with increasing abdominal discomfort. A nasogastric tube was placed but no fluid or air was obtained. Since no suspicion regarding the nasogastric tube's place was araised, chest x-ray was not obtained. The patient gradually developed hemodynamic instability and was treated with crystalloid transfusions but her blood pressure dropped continuously to 70/40 mmHg and her pulse rate increased to 140 beats/min. The ECG showed no signs of ischemia and her hematocrit was 28%. The patient was re-intubated and mechanicaly ventilated with great difficulty. At a Vt of 550 ml, a rate of 12/min, with a peak airway pressure of 42 cmH2o and mean airway pressure of 22 cmH2o. Her condition deteriorated rapidly, with no response to crystalloid and blood transfusion, and aggressive use of vasoactive agents. The abdomen was massively distended despite the use of muscle relaxants and the patient became anuric. IAP measured via a Foley bladder catheter according to the modified Kron technique described by Malbrain et al. [12], was 31 mmHg. As the patient presented a life threatening condition, she was taken immediately for re-exploration without any further diagnostic or therapeutic measures. She underwent laparotomy which revealed a minimal amount of intraperitoneal fluid but an enormously distended stomach. The nasogastric tube was situated in a small sliding hernia and did not drain the distended stomach. The nasogastric tube was flushed and re-positioned, allowing the deflation of the stomach. After deflation, the patient's condition immediately improved. The blood pressure rose to 140/70 mmHg, the heart rate decreased to 104/min, and the urine output increased to 300 ml/hr. Vasopressor agents were stopped. The peak airway pressure dropped to 21 cmH_2_O and the mean airway pressure dropped to 8 cmH_2_O. The repeated postoperative IAP measurements were within the normal limits. The postoperative course was complicated by line sepsis, treated by line removal, broad-spectrum antibiotics and vasoactive agents with a favorable response. The patient was discharged from hospital after 10 days in good condition.

## Discussion

The World Society of the ACS (WSACS) has recently developed consensus definitions outlining standards for IAP measurement as well as diagnostic criteria for IAH and ACS based upon both the best available clinical evidence and expert opinion.

Acute and rapid elevation in intra-abdominal pressure exceeding 12 mmHg is considered to be pathologically elevated and has been termed intra-abdominal hypertension [10]. IAH is graded as follows: grade I: IAP 12–15 mmHg, Grade II: IAP 16–20 mmHg, Grade III: IAP 21–25 mmHg, and grade IV: IAP > 25 mmHg.

ACS is defiened as a sustained IAP > 20 mmHg (with or without an APP < 60 mmHg) that is associated with new organ dysfunction or failure [13,14].

The phrase abdominal compartment syndrome was first used by Kron et al. [12] in the early 1980s to describe the physiologic effects of intra-abdominal hypertension complicating a ruptured aortic aneurysm surgery. However, most of our knowledge about this entity has evolved over the past few years [4,5,15].

ACS can develop in both nonsurgical and surgical patients, either preoperatively or postoperatively. Although the incidence of the ACS was found to range between 5% and 15% of trauma patients [16], an increased IAP (18 mmHg) was observed in up to 41% of surgical patients [17]. ACS is most commonly diagnosed in patients sustaining abdominal or pelvic trauma, or suffering some other intra-abdominal hemorrhagic catastrophe. Less common etiologic factors include retroperitoneal hematoma or edema, bowel obstruction, ascites, and necrotizing pancreatitis [4,18].

Sustained elevation of intra-abdominal pressure causes increased intrathoracic pressure and abnormalities in pulmonary dynamics, increased afterload, decreased venous return, decreased cardiac output, and decreased perfusion to the kidneys and intestinal mucosa [19-21].

Early recognition and prompt intervention are essential to optimize a patient's outcome. The management and treatment of ACS is not difficult once the diagnosis is considered, but diagnosis is many times obscured in critically ill or shocked patients.

Therefore, a high index of suspicion is needed in any patient who acutely develops tense abdominal distention combined with one or more of the following: decreased cardiac output, oliguria progressing to anuria, and respiratory failure with high ventilatory pressures.

The diagnosis of IAH and ACS is dependent upon the accurate and frequent measurement of IAP. Recent prospective studies that evaluated the factors evolving IAH and ACS suggested that there is no specific type of patient or disease or treatment that reliable indicates when IAP needs to be measured, or when the measurement is not necessary in a mixed ICU population. Therefore, IAP should be routinely measured, for the initial overall evaluation of ICU patients [3].

According to the consensus recommendations of the World Society of the ACS, a baseline IAP measurement should be obtained if two or more risk factors for IAH/ACS are present, and serial IAP measurements should be performed throughout the patient's critical illness, if IAH is present [14].

To the best of our knowledge, no case of postoperative gastric dilatation causing ACS has been described. Mook et al. [22] described a case of ACS caused by distended stomach due to duodenal ulcer bleeding.

## Conclusion

Deterioration of the patients' condition following a major abdominal surgery should raise a suspicion of gastric dilatation, especially in cases of distended abdomen, oliguria and increased airway pressures. Postoperative untreated gastric dilatation may cause abdominal compartment syndrome. Measurement of IAP should be done promptly in these patients. When increased IAP is present, the correct placement of the nasogastric tube must be carefully confirmed to avoid unnecessary surgery.

## Competing interests

The author(s) declare that they have no competing interests.

## Authors' contributions

All the authors have been involved in literature search, writing and approval of final manuscript.
